# Special Protein or RNA Molecules Computational Identification

**DOI:** 10.3390/ijms241411312

**Published:** 2023-07-11

**Authors:** Ren Qi, Quan Zou

**Affiliations:** 1 Yangtze Delta Region Institute (Quzhou), University of Electronic Science and Technology of China, Quzhou 324000, China; qiren@tju.edu.cn; 2 School of Life Science and Technology, University of Electronic Science and Technology of China, Chengdu 610054, China; 3 Institute of Fundamental and Frontier Sciences, University of Electronic Science and Technology of China, Chengdu 610054, China

The identification of special protein or RNA molecules via computational methods is of great importance in understanding their biological functions and developing new treatments for diseases. Computational methods can help identify proteins and RNA molecules by analyzing genomic and proteomic data, as well as using machine learning algorithms and other computational techniques to identify patterns and structures within the data. The identification of special proteins or RNA molecules can lead to the discovery of new therapeutic targets for the treatment of diseases, such as cancer or viral infections. By understanding the structures and functions of these molecules, researchers can design drugs that target them specifically, resulting in more effective treatments with fewer side effects. Furthermore, computational methods can also aid in the identification of biomarkers, which are molecules that indicate the presence of a disease. By identifying specific proteins or RNA molecules that are associated with a particular disease, researchers can develop diagnostic tests that can detect the disease at an early stage, leading to earlier treatment and improved patient outcomes. 

Non-coding RNAs (ncRNAs) are RNA molecules that do not encode proteins but have regulatory roles in gene expression. Proteins are essential macromolecules that carry out a variety of functions in a cell, including catalyzing biochemical reactions, transporting molecules, and regulating gene expression. Some good work has been published on the role of molecular dynamics simulations in understanding the functions of RNA-based systems [1,2]. In this Special Issue, we encourage authors to pay attention to non-coding RNA molecules, proteins, RNA function, and the RNA–disease relationship. Some network methods, including random walk and matrix factorization, have been employed in RNA–disease relationship prediction before; however, they are not robust. We are pleased to state that more novel, robust methods and gold benchmark datasets are presented in this Special Issue.

The interactions between ncRNAs and proteins are critical for many biological processes, including transcriptional and post-transcriptional regulation, RNA splicing, RNA editing, translation, and protein stability. Predicting interactions between ncRNAs and proteins is crucial to understanding the molecular mechanisms of these processes and identifying potential targets for drug development. Zhan et al. predicted ncRNA–protein interactions based on an innovative as well as practical deep learning model and improved sequence information, and they employed a stacked autoencoder network to extract hidden high-level features [3]. Zhan’s method for predicting the interactions between ncRNAs and proteins complemented experimental techniques and provided insights into the roles of ncRNAs and proteins in cellular processes.

Predicting disease-related microRNAs (miRNAs) is an important area of research with significant potential for improving our understanding of disease mechanisms and developing new therapies for a wide range of diseases. miRNAs are small non-coding RNA molecules that play important roles in regulating gene expression in a variety of biological processes. There are several methods for predicting disease-related miRNAs, including computational approaches that analyze gene expression data, sequence information, and other genomic features. In this Special Issue, Xuan et al. proposed a dual convolutional neural-network-based method to predict candidate disease-related miRNAs and captured the topology structures of the miRNA and disease networks [4]. The method can identify miRNAs that are differentially expressed in diseased tissues, as well as those that are predicted to target genes involved in disease pathways. 

Circular RNAs (circRNAs) are a type of non-coding RNA that have been found to play important roles in various biological processes, including gene regulation, transcriptional and post-transcriptional control, and the development of certain diseases. As a result, the detection of circRNAs from RNA-seq data has become an important research topic in molecular biology. One of the main challenges in detecting circRNAs from RNA-seq data is their circular structures, which make them resistant to traditional RNA sequencing approaches; however, recent advances in RNA sequencing technology and bioinformatics tools have made it possible to detect circRNAs from RNA-seq data. Zhang et al. provided a novel computing method, CIRCPlus, that located circRNA candidates by identifying a set of back-spliced junction reads by comparing the local similar sequence of each pair of spanning junction reads [5]. Zhang et al. showed that circRNAs play important roles in the regulation of gene expression. They provided insights into the regulation of gene expression, the development of certain diseases, and the complex regulatory networks that control biological processes.

Protein function prediction and identification are crucial for the development of new biotechnologies. For example, identifying proteins with specific functions can lead to the development of new enzymes for industrial processes. Seven papers focus on describing protein function prediction or protein identification, which include the prediction of signal peptides in proteins, protein hydroxylation site prediction, protein–protein interaction (PPI) prediction, and protein identification. To identify signal peptides in proteins from malaria parasites, Burdukiewicz et al. present a prediction method called signalHsmm [6]. The proposed method applied a hidden semi-Markov model, combined with the structure of signal peptides and the physicochemical properties of amino acids. Long et al. designed a hybrid deep learning model integrating the convolutional neural network (CNN) and long short-term memory network (LSTM) to predict protein hydroxylation sites [7]. Liu et al. focused on intrinsically disordered protein or region identification in the MobiDB database and the DisProt database based on conditional random fields [8]. They incorporated sequence-based features, including position-specific scoring matrices, kmer, predicted secondary structures, and relative solvent accessibilities, and the results showed that the proposed method outperformed 25 existing methods. Li et al. proposed a new sequence-based method, which combined a relevant vector machine model with low-rank matrix approximation, to predict protein–protein interactions [9]. Furthermore, in terms of protein identification, Xu et al. concentrated on the study of antioxidant protein identification; they proposed a machine learning method, SeqSVM, to predict antioxidant proteins through extracted sequence features [10]. Notably, our Special Issue provided two new web servers regarding protein prediction: Niu et al. created a web server, RFAmyloid, to predict amyloid proteins [11]. The predictor incorporated random forest and SVMProt 188-D feature extraction methods to identify amyloids. In addition, Pan et al. provided a web server, PhagePred, and they employed a multinomial naive Bayes with a g-gap feature tree to identify bacteriophage virion proteins [12]. Protein function prediction and identification advanced our understanding of fundamental biological processes. By studying proteins and their functions, researchers can learn more about how cells work and how diseases develop.

Overall, papers in this Special Issue have demonstrated that the identification of special proteins or RNA molecules via computational methods is a powerful tool that can help advance our understanding of biological processes and lead to the development of new therapies and diagnostic tools. We expect that such studies will receive great attention. Finally, we thank all of the efforts of the authors, reviewers, and staff at the *International Journal of Molecular Sciences* Editorial Office.
